# Cross-modal Action Complexity: Action- and Rule-related Memory Retrieval in Dual-response Control

**DOI:** 10.3389/fpsyg.2017.00529

**Published:** 2017-04-07

**Authors:** Aleks Pieczykolan, Lynn Huestegge

**Affiliations:** Department of Psychology, University of WürzburgWürzburg, Germany

**Keywords:** dual-response costs, cross-modal action, oculomotor control, task rules, dual tasks

## Abstract

Normally, we do not act within a single effector system only, but rather coordinate actions across several output modules (cross-modal action). Such cross-modal action demands can vary substantially with respect to their complexity in terms of the number of task-relevant response combinations and to-be-retrieved stimulus–response (S–R) mapping rules. In the present study, we study the impact of these two types of cross-modal action complexity on dual-response costs (i.e., performance differences between single- and dual-action demands). In Experiment 1, we combined a manual and an oculomotor task, each involving four response alternatives. Crucially, one (unconstrained) condition involved all 16 possible combinations of response alternatives, whereas a constrained condition involved only a subset of possible response combinations. The results revealed that preparing for a larger number of response combinations yielded a significant, but moderate increase in dual-response costs. In Experiment 2, we utilized one common lateralized auditory (e.g., left) stimulus to trigger incompatible response compounds (e.g., left saccade and right key press or vice versa). While one condition only involved one set of task-relevant S–R rules, another condition involved two sets of task-relevant rules (coded by stimulus type: noise/tone), while the number of task-relevant response combinations was the same in both conditions. Here, an increase in the number of to-be-retrieved S–R rules was associated with a substantial increase in dual-response costs that were also modulated on a trial-by-trial basis when switching between rules. Taken together, the results shed further light on the dependency of cross-modal action control on both action- and rule-related memory retrieval processes.

## Introduction

In our daily life, we are used to do several things at the same time, that is, we routinely execute multiple actions simultaneously. In cognitive psychology, there is a long research tradition in which the underlying mechanisms of such situations are unraveled. In this context, two closely related research fields can be distinguished: Research on *multitasking* (specifically *dual-tasking*) and research on *multiple-action* control. Dual tasking necessarily involves the simultaneous processing of two tasks, that is, two independent streams of processing triggered by two distinct stimuli or stimulus characteristics, but irrespective of the need to finally produce (at least) two overt responses (e.g., one task may involve memorization only). In contrast, research on multiple-action control can be regarded as narrower in the sense that it only subsumes situations in which two or more responses are overtly executed. At the same time, however, it can also be regarded as broader in the sense that it also covers situations in which *one aspect of a stimulus* defines the selection and execution of a *dual-response compound* consisting of two discriminable responses (Holender, 1980; Fagot and Pashler, 1992). Since most theories underlying the control of multiple actions were developed in the context of dual-task studies, it is important to examine whether and to which extent underlying concepts can also be transferred to those situations that involve multiple-action control but do not represent a typical dual-task situation involving two independent stimuli. In the present study, we are utilizing both approaches in two experiments (i.e., triggering two responses with (a) two separate stimuli and (b) one single stimulus) to focus on the role of action- and rule-related memory retrieval processes in multiple-action control involving distinct effector systems (i.e., oculomotor and manual responses).

Previous dual-task research has focused on explaining how two simultaneous task processing streams can interfere with each other. To define each task, instructions – in form of a set of stimulus-response rules – are explicitly presented to participants at the beginning of the experiment. Representations of these rules in working memory allow participants to correctly bind responses to stimuli in each trial, ensuring task-appropriate action (Logan and Gordon, 2001). Working memory is typically defined as a cognitive system responsible for maintenance, updating, and manipulation of task-relevant information (e.g., Baddeley and Hitch, 1974; Daneman and Carpenter, 1980; Baddeley et al., 2011). In the context of multiple-action control, working memory is thus necessary for maintaining task-relevant representations of stimuli and responses, and should also provide the basis for correctly binding stimuli and responses according to task rules. As such, it is regarded as an integral component for executive control in dual-task frameworks (e.g., Meyer and Kieras, 1997). Note that the well-known storage limitations of working memory render it impossible to maintain simultaneous representations of all potentially task-relevant stimuli, responses, and binding rules, thus calling for retrieval processes (e.g., in terms of transferring pre-activated long-term memory representations into the focus of attention in working memory, see Cowan, 1995, 2016; Mayr and Kliegl, 2000; Oberauer, 2002; Oberauer et al., 2013). In this way, response selection in multiple action control can be conceptualized as the retrieval of the correct (i.e., rule-appropriate) response (among other response alternatives) in each task based on task rules that have been correctly retrieved among potential alternative rules (see also Verbruggen et al., 2014).

Interestingly, working memory mechanisms that are specific for the coordination of multiple-action demands have only seldom been addressed explicitly (see Hazeltine and Wifall, 2011, for a detailed discussion on this issue). For example, Hommel (1998a) demonstrated that features (e.g., spatial codes) of a secondary task response, that is, a response in a Task 2 that was executed after a Task 1 response, determined the speed of the primary Task 1 response [*backward crosstalk effect* based on spatial response–response (R–R) compatibility]. This effect (which is based on conflict between two task-appropriate response representations) is difficult to explain when assuming that response selection of Task 1 must be finished before any response-related processing for Task 2 occurs (i.e., within a serial response selection bottleneck account, see Pashler, 1994). To explain the backward crosstalk effect, it has been discussed whether representations of the task rules for Task 2 in working memory might already be active during Task 1 processing (due to partially automatic S–R bindings in form of *memory event files*, see Hommel, 1998a,b). In this way, response activation in Task 2 can prime or interfere (in the case of compatible or incompatible response codes, respectively) with response-related processing in Task 1 (see Hommel and Eglau, 2002; Ellenbogen and Meiran, 2008, 2010, for in-depth discussions). Note, however, that while the present study on a general level also addresses the interaction of multiple-action selection and memory processes, the specific focus of the present study is somewhat different: Instead of analyzing backward crosstalk effects in dual tasks, we measure dual-response coordination efficiency as indexed by dual-response costs (see below for details) and focus on retrieval competition between currently appropriate and *in*appropriate representations within a trial.

As outlined above – and in contrast to typical dual-task settings – multiple-action control does not necessarily involve two distinct task processing streams in form of separate response selection processes. One specific example is the case of dual-response compounds in which two responses are triggered by the same aspect of a stimulus. Thus, interference within such dual-response compounds cannot be readily explained by mechanisms referring to interference between independent rules (and separate response selection processes) for Task 1 and Task 2. As a response to this issue, Huestegge and Koch (2010; see also Huestegge, 2011, for an extended version) suggested an alternative framework of multiple-action control that does not involve two distinct response selection processes (one for each independent task) within a trial, but instead suggests one common “mapping selection” stage in which feature codes (e.g., spatial codes) are bound to task-relevant effector codes in accordance with task instructions. If, for example, a left auditory stimulus indicates the execution of a response compound consisting of a leftward saccade and a right manual key press, it is assumed that the mapping selection stage involves the implementation of a corresponding binding pattern among codes, that is, the binding of a “left” spatial code with the “saccade” effector code and of a “right” spatial code with a “manual” effector code. Thus, such a binding pattern specifies the required response compound (or response combination). The model also involves further assumptions. For example, more complex binding patterns (those involving more and/or potentially conflicting codes) are assumed to take more time (e.g., when two spatial codes instead of one need to be bound to respective effector systems). Finally, the model assumes that *memory-based conflict* between task-relevant binding patterns can occur in terms of retrieval competition. Specifically, persisting activation of a binding pattern from the previous trial is assumed to interfere with selecting a different binding pattern in the current trial (retrospective interference, equivalent to response repetition/switch effects in single task control, see Bertelson, 1965; see also Janczyk, 2016, for between-trial modulations of the backward crosstalk effect in dual tasks). Additionally, and more relevant for the present study, we assumed that all task-relevant binding patterns are activated to some extent (i.e., prepared) and thus held in memory based on task instructions (e.g., see Pfeuffer et al., 2017, on explicit rule implementation). As a result, this baseline activation of all potentially upcoming binding patterns should impact on each individual mapping selection in a current trial and make it more difficult to coordinate both responses simultaneously. A clear prediction of this assumption is that any increase in the number of task-relevant binding patterns should negatively affect dual-response coordination efficiency, which in the present study is defined as an inverse measure of dual-response costs, that is, the additional time to execute the same response in a response compound (i.e., in dual-response condition) than in isolation (i.e., in single-response condition). This prediction of the model by Huestegge and Koch (2010) and Huestegge (2011) has not been directly addressed yet in previous research on multiple-action control, and will be tested in Experiment 1 of the present study.

A related open issue (although not directly associated with predictions from our model) is the impact of the to-be-memorized stimulus-response binding rules on dual-response coordination efficiency in multiple-action control. While the number of task rules was shown to affect backward crosstalk effects in dual tasks (see Hommel and Eglau, 2002; Ellenbogen and Meiran, 2008, 2010), the question of how the number of instructed task rules affects dual-response coordination efficiency in response compound control (where a single stimulus defines both responses) is still an open issue. Experiment 2 of the present study will address this issue in order to further specify the potential interactions between memory (here: related to the number of task rules) and multiple-action control.

Across both experiments, we thus study the impact of response binding pattern retrieval (by manipulating the number of task-relevant response binding patterns while keeping the amount of S–R rule sets constant; Experiment 1) and rule retrieval (by manipulating the number of task-relevant rule sets while keeping the number of task-relevant response binding patterns constant; Experiment 2). Both manipulations have in common that they are associated with an increase/decrease of the complexity of memory demands (i.e., the amount of retrieval competition) in multiple-action control. Specifically, we focus on effects of these factors on dual-response coordination efficiency (see above). Note that this current focus on dual-response costs as a dependent measure differs substantially from just analyzing effects on overall RTs in each effector system, because absolute RT levels reflect more basic phenomena that are not necessarily specific for *multiple*-action control. In contrast, dual-response costs are typically regarded as an index of dual-response interference (e.g., Navon and Miller, 1987; Schumacher et al., 2001; Huestegge and Koch, 2009), and as such should reflect the ability (or efficiency) to coordinate two responses as a function of the complexity of memory demands. Following a research tradition in our lab (Huestegge and Koch, 2009, 2010; Huestegge and Adam, 2011; Pieczykolan and Huestegge, 2014), we focused on cross-modal action demands involving both oculomotor and manual actions. We considered this combination of effector systems particularly interesting, since previous research has suggested different underlying control characteristics as a function of response selection difficulty for the two effector systems (e.g., manual responses follow Hick’s law while oculomotor responses do not, see Kveraga et al., 2002).

## Experiment 1

In Experiment 1, we combined a manual and an oculomotor task, each involving four response alternatives. Specifically, we decided to vary the overall number of binding patterns by manipulating the number of response alternatives in the oculomotor response (while keeping manual response alternatives constant). Therefore, any differences in dual-response costs for the manual response can only be attributed to the specific influence of the dual-response condition and not to a difference of the number of response alternatives in single-response conditions. Both types of responses (manual and oculomotor) were triggered by separate stimulus features. Crucially, one (unconstrained) condition involved all 16 (4^∗^4) possible combinations of response alternatives (i.e., of binding patterns), whereas a constrained condition only involved a subset of combinations (i.e., 8) to manipulate the number of relevant cross-modal response binding patterns. Specifically, in the constrained condition we limited the range of oculomotor response alternatives from four target positions to two target positions. This was implemented to focus the analysis on the manual responses, for which all aspects of the design are comparable regarding the number of response alternatives (i.e., 4) and which exhibit the larger amount of dual-response costs (based on previous studies of this response combination, see Huestegge and Koch, 2009, 2010, 2013; Pieczykolan and Huestegge, 2014) and which therefore should be more sensitive to manipulations affecting dual-response situations. As outlined in the introduction and based on the framework by Huestegge and Koch (2010; see also Huestegge, 2011) we tested the hypothesis that manual dual-response costs are larger in the unconstrained (vs. constrained) response pattern condition, which would suggest that the number of task-relevant mapping patterns stored in memory affects dual-response coordination efficiency.

### Method

#### Participants

Forty-eight participants were randomly assigned to two groups (unconstrained vs. constrained binding patterns group). The mean age was 24.6 years in the unconstrained group (*SD* = 3.7, range = 19–33, nine male) and 23.8 years in the constrained group (*SD* = 4.1, range = 17–34, four male). All participants gave informed consent and received monetary reimbursement or course credits for participation.

#### Apparatus and Stimuli

**FIGURE 1:**
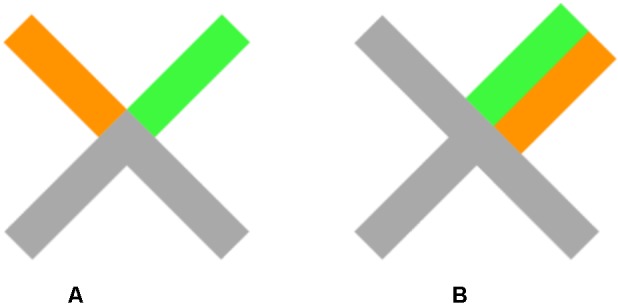
**Examples of a central stimulus in the incompatible condition (A)** and compatible condition **(B)** in Experiment 1.

Participants were seated in front of a standard 21″ CRT screen. Eye movements of the right eye were recorded at a sampling rate of 1000 Hz using an Eyelink 1000 eye tracker (SR Research, Ottawa, ON, Canada). On a black background, a gray central fixation cross (30 px × 30 px in X shape, see **Figure 1**) as well as four gray rectangular saccade targets (squares with an edge length of 20 px) located at 9.4° diagonally at the upper left, upper right, lower left, and lower right remained present throughout. As manual response keys, four keys (in a square-like spatial arrangement) from the standard keyboard were chosen (upper left, upper right, lower left, and lower right key) and marked with gray stickers. The visual stimuli were represented as color changes of one line of the limbs of the central fixation cross (**Figure 1**). For example, an eye movement to the upper left target combined with a manual response with the upper right key was indicated as an orange limb pointing toward the corresponding saccade target and a green limb indicating the corresponding manual key. In the case of compatible saccade and manual response demands (e.g., both “upper right”), one limb of the central “X” was half green and half orange but of the width of two limbs.

#### Procedure

Each trial started with the presentations of the central fixation cross (400 ms) which then changed partially in color to represent the visual imperative stimulus (with a duration of 350 ms). Participants were instructed to either move their gaze to the spatially compatible square on the screen (single saccade blocks), to press the compatible key (left/right index fingers and thumbs operating the four keys in the manual task), or to do both (dual-response blocks) as fast and accurately as possible. While in the unconstrained group all combinations of manual and oculomotor responses were possible, thus all 16 binding patterns were present, in the constrained group the range of potential saccade alternatives was reduced to two resulting in a reduction of the number of total response binding patterns (8 in total). However, participants were not explicitly informed about this constraint, and all four saccades targets were still visible in the constrained group in order to obtain a comparable visual stimulus display. In conditions that required saccades (saccade response in single and dual-response blocks), subjects were instructed to return to the central fixation cross after responding. Each participant completed nine blocks in total consisting of three sequences of the experimental blocks (single manual, single saccade, dual). The order within the sequences was counterbalanced across participants but was constant within participants (e.g., one participant completed the sequence “manual, dual, saccade” three times). Within each block, 48 stimuli were presented in random order with an inter-stimulus interval of 2500, 3000, or 3500 ms that was counterbalanced across all instances of binding patterns. Prior to each block, subjects underwent a calibration routine.

#### Design

Each effector (manual, saccade) was analyzed separately with the main focus on effects on the (comparable) manual responses. Response condition (single vs. dual response) was manipulated within-participants while the number of response patterns (constrained vs. unconstrained) was varied between-participants. The order of single-response blocks and dual-response blocks as well as the color-effector assignment were counterbalanced across participants. Dependent variables were RTs and error rates (response omissions/wrong response targets).

### Results and Discussion

One participant in the unconstrained group was excluded from the analyses because of extraordinary high error rates in several conditions (>60%). Thus, the final analysis refers to 23 participants in the unconstrained group and 24 participants in the constrained group. Because the number of response alternatives varied only for saccade responses, we calculated two separate ANOVAs for saccades and manual responses. RT analyses were performed on correct trials only, while trials with erroneously executed saccades in single manual condition were considered invalid and therefore excluded from the analysis (2.1% of the collected data). Additionally, we excluded compatible trials (i.e., those trials in which both responses were directed toward the same direction) from the analysis (25% of the dual-response trials; see Appendix for an analysis of R–R compatibility effects).

#### Manual Responses

**FIGURE 2:**
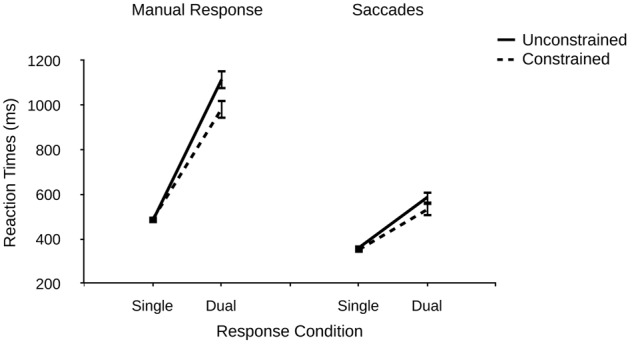
**Reaction times (ms) for manual responses and saccades as a function of response condition (single vs. dual) and number of binding patterns (unconstrained vs. constrained) in R–R incompatible trials in Experiment 1.** Error bars denote standard errors.

A mixed 2 (response condition) × 2 (group) ANOVA revealed a significant main effect of response condition on manual RTs, *F*(1,45) = 473.08, *p* < 0.001, $\eta_{p}^{2}$ = 0.913, indicating longer RTs in dual- vs. single-response conditions (1050 ms vs. 488 ms). This finding replicates many previous reports of manual response sensitivity to additional oculomotor response demands (e.g., Huestegge and Koch, 2009, 2010, 2013; Huestegge, 2011; Pieczykolan and Huestegge, 2014). There was no significant main effect of the number of binding patterns, *F*(1,45) = 2.57, *p* = 0.116. Importantly, however, there was a significant interaction of response condition and the number of binding patterns, *F*(1,45) = 7.15, *p* = 0.010, $\eta_{p}^{2}$ = 0.137, indicating larger dual-response costs for manual responses in the unconstrained vs. constrained group (619 ms vs. 496 ms, see **Figure 2**). Thus, the main hypothesis of Experiment 1 was confirmed by the data: A larger number of task-relevant binding patterns increases dual-response interference and thus decreases dual-response coordination efficiency, most likely due to greater retrieval competition between binding patterns. This result clearly demonstrates that dual-response coordination efficiency is not simply determined by the number of response alternatives for the individual tasks (which was held constant). Probably, even though responses were triggered by separate stimuli, in dual-response conditions the representation of the number of response alternatives for the saccade response “spilled over” into that for the manual responses (which are known to be susceptible to manipulations of response alternatives), and this crosstalk-like effect may have elevated RTs in the unconstrained condition compared with the constrained condition.

Based on the same data as in the manual RT analysis, we also analyzed *error rates* to rule out a speed-accuracy tradeoff (in terms of reversed result patterns in the error data) as a potential alternative explanation. However, the data pattern did not support the notion of speed-accuracy tradeoffs (**Table 1**). While we observed a significant main effect of response condition, *F*(1,45) = 32.87, *p* < 0.001, $\eta_{p}^{2}$ = 0.422, indicating a greater manual error rate in dual- vs. single-response conditions (9.6% vs. 2.6%), we observed neither a significant main effect of the number of response patterns, *F* < 1, nor a significant interaction, *F* < 1.

**Table 1:** Error rates (%) for manual responses and saccades in R–R incompatible trials as a function of number of binding patterns (constrained vs. unconstrained), and response condition (single and dual) in Experiment 1.

Number of binding patterns	Constrained		Unconstrained	
Response condition	Single	Dual	Single	Dual
Manual responses	2.9 (0.7)	10.3 (1.5)	2.3 (0.7)	8.8 (1.6)
Saccades	2.1 (0.4)	11.8 (1.4)	1.7 (0.5)	13.5 (1.5)

*Numbers in parentheses denote standard errors.*

#### Saccades

An analysis analog to that for manual responses was conducted for saccade responses. There was a significant main effect of response condition, *F*(1,45) = 144.29, *p* < 0.001, $\eta_{p}^{2}$ = 0.762, indicating longer saccade RTs in dual- vs. single-response conditions (561 ms vs. 357 ms), replicating previous reports of saccade response sensitivity to additional manual response demands (e.g., Huestegge and Koch, 2009, 2010, 2013; Huestegge, 2011; Pieczykolan and Huestegge, 2014). There was no significant effect of the number of binding patterns, *F*(1,45) = 1.24, *p* = 0.271, and no significant interaction, *F*(1,45) = 1.85, *p* = 0.180.

The analysis of *saccade errors* revealed a significant main effect of response condition, *F*(1,45) = 107.17, *p* < 0.001, $\eta_{p}^{2}$ = 0.704, indicating a greater saccade error rate in dual- vs. single-response conditions (12.7% vs. 1.9%). However, there was neither a significant main effect of the number of binding patterns, nor a significant interaction, both *F*s < 1.

## Experiment 2

In Experiment 2, we aimed at studying the effects of the number of task-relevant rule sets stored in memory on dual-response coordination efficiency, while keeping the number of task-relevant response combinations (i.e., binding patterns) constant. We used one common lateralized auditory stimulus (presented either to the left or right ear) to trigger incompatible response compounds (e.g., a left saccade and a right key press, or vice versa). Instead of four response alternatives (as in Experiment 1), there were only two response alternatives for each effector system (left/right saccade and left/right key press). Response demands across effector systems were always spatially incompatible (see also Huestegge and Koch, 2010; Pieczykolan and Huestegge, 2014), resulting in only two possible response compounds (i.e., two binding patterns) in this experiment (saccade left + manual key press right and saccade right + manual key press left). Using only incompatible response demands allowed us to manipulate the number of task rule sets (both task rule sets being of similar difficulty) without changing the number of task-relevant response compounds: Crucially, while one condition only involved *one* set of task-relevant rules, another condition involved *two* sets of task-relevant S–R rules (coded via auditory stimulus type: noise vs. tone). For example, in the *one S–R rule* condition a tone signaled an S–R compatible saccade and an S–R incompatible key press. Consequently, a tone on the left required a leftward (compatible) saccade and a right (incompatible) key press while a tone on the right required a compatible (right) saccade and an incompatible (left) key press (one rule set: saccade compatible, manual incompatible). In the *two S–R rule* condition, both stimulus types were presented in an intermixed manner (two rule sets: tone saccade compatible, manual incompatible, noise saccade incompatible, manual compatible). Crucially, this resulted in the situation that the same response binding pattern (e.g., saccade left + manual key press right) could be triggered by two different stimuli (e.g., a tone on the left or a noise burst on the right). Note that unlike in Experiment 1, the number of response binding patterns (2) remained constant throughout the experiment. Therefore, if only the number of relevant response binding patterns determined the efficiency of dual-response control, we should expect similar dual-response costs in the one-rule vs. two-rule condition. However, if the number of S–R rule sets (and the associated rule retrieval from memory) affected dual-response control efficiency, we should observe substantially greater dual-response costs in the two-rule (vs. one-rule) condition.

### Method

#### Participants

Twenty-four new participants (mean age = 23.61 *SD* = 4.42, range = 19–41, 20 female) with normal or corrected-to-normal vision were tested. They gave informed consent and received course credits or monetary reimbursement for participation.

#### Apparatus and Stimuli

An Eyelink II was utilized as eye-tracking device. The central fixation cross consisted of a green plus sign, and two saccade targets (at 8° to the left and right of the fixation cross) were presented in form of two green squares (1/3° each), which remained present throughout. Different to Experiment 1, we used auditory stimuli consisting of lateralized harmonic tones (with a fundamental frequency of 400 Hz mixed with 800 and 1200 Hz) and pink noise bursts (both with a duration of 50 ms) that had equal loudness and were presented via headphones.

#### Procedure

In each trial, an auditory stimulus was presented to the left or right ear. Participants were instructed to respond as fast and accurately as possible either by moving their gaze to a square on the screen (saccade response in single blocks), pressing a key (left/right index fingers operating two keys with a distance of 30 cm from the bottom row of a standard keyboard), or both (dual-response blocks). In the dual-response blocks, both responses were instructed to be executed spatially incompatible to each other. That is, there were only two response compounds in this experiment (saccade left + manual key press right and saccade right + manual key press left). Crucially, while the *one-rule condition* only involved one set of single task-relevant S–R rules (e.g., tone compatible saccade + incompatible manual response), the *two-rule* condition involved two opposing sets of task-relevant S–R rules (each set of rules coded via a respective auditory stimulus type: noise vs. tone; e.g., tone compatible saccade + incompatible manual response, noise incompatible saccade + compatible manual response). Thus, in one condition there was only one stimulus type (only tone or only noise), while the other condition involved both stimulus types (tone and noise).

The specific S–R assignments of stimulus types to response compounds was constant within participants and counterbalanced across participants. Each participant completed 12 blocks consisting of 36 trials each. Within each block, stimuli to the left and right were presented in randomized sequence with a response-stimulus interval of 1500, 2000, or 2500 ms. Prior to each block, subjects underwent a calibration routine.

#### Design

Due to comparable demands in both effector systems, effector modality was included here as a factor in the analysis. Thus, the within-subject variables were modality (saccade vs. manual response), response condition (single vs. dual), and the number of S–R rule sets (one vs. two). The order of single-response blocks and the two types of dual-response blocks (one S–R rule set vs. two S–R rule sets) were counterbalanced across participants. Dependent variables were RTs and error rates.

### Results and Discussion

**FIGURE 3:**
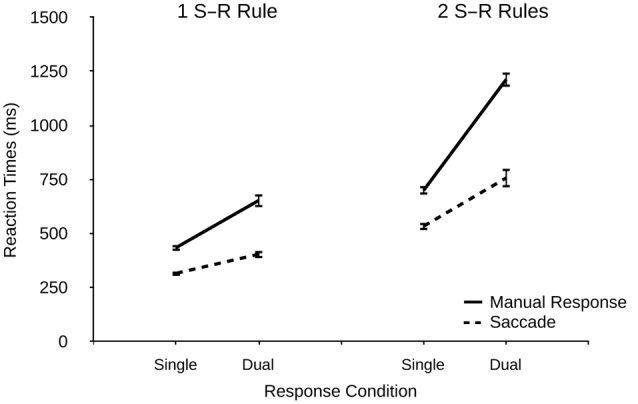
**Reaction times (ms) for manual responses and saccades as a function of response condition (single and dual) and number of S–R rule sets (1 vs. 2) in Experiment 2.** Error bars denote standard errors.

Two participants were excluded due to extraordinary high error rates (>60%). Response times are depicted in **Figure 3** and error rates are shown in **Table 2**. RT analyses included only correct trials. Trials with erroneously executed saccades in single manual condition were considered invalid and therefore excluded from the analysis (1.6% of the collected data).

**Table 2:** Error rates (%) for manual responses and saccades as a function of task condition (single vs. dual) and number of S–R rule sets in Experiment 2.

	1 S–R Rule		2 S–R Rules	
	Single response	Dual response	Single response	Dual response
Manual responses	1.7 (0.4)	3.4 (0.9)	9.7 (2.5)	17.1 (3.4)
Saccades	7.7 (2.4)	11.7 (2.7)	15.9 (3.5)	23.7 (3.9)

*Numbers in parentheses denote standard errors.*

#### Response Times

There was a significant effect of response modality on RTs, *F*(1,21) = 206.873, *p* < 0.001, $\eta_{p}^{2}$ = 0.908, indicating faster RTs for saccades vs. manual responses (503 ms vs. 703 ms). There was also a significant main effect of response condition, *F*(1,21) = 226.44, *p* < 0.001, $\eta_{p}^{2}$ = 0.915 (single: 497 ms, dual: 757 ms), and a significant main effect of the number of S–R rule sets, *F*(1,21) = 265.93, *p* < 0.001, $\eta_{p}^{2}$ = 0.927 (one S–R rule: 452 ms, two S–R rules: 801 ms). Thus, manipulating the number of S–R rule sets had a very pronounced effect on overall performance.

There was a significant interaction of modality and response condition, *F*(1,21) = 95.60, *p* < 0.001, $\eta_{p}^{2}$ = 0.820, indicating larger dual-response costs for manual responses than for saccades (365 ms vs. 156 ms). There was also a significant interaction of modality and the number of S–R rule sets, *F*(1,21) = 52.54, *p* < 0.001, $\eta_{p}^{2}$ = 0.714, suggesting a stronger effect of the number of S–R rule sets in manual responses than in saccades (413 ms vs. 284 ms). Most importantly, and in line with our prediction, there was a significant interaction of response condition and the number of S–R rule sets, *F*(1,21) = 33.83, *p* < 0.001, $\eta_{p}^{2}$ = 0.617, indicating greater dual-response costs when two (vs. one) S–R rule sets were present (368 ms vs. 153 ms), thus demonstrating that despite the same number of response alternatives the number of rule sets strongly contributed to dual-response efficiency. Finally, the three-way interaction was significant, *F*(1,21) = 37.18, *p* < 0.001, $\eta_{p}^{2}$ = 0.639, indicating that the effect of greater dual-response costs under two (vs. one) S–R rules was more pronounced for manual responses (513 ms vs. 218 ms) than for saccades (223 ms vs. 87 ms).

#### Error Rates

Error rates are shown in **Table 3**. There was a significant effect of response modality on error rates, *F*(1,21) = 18.58, *p* < 0.001, $\eta_{p}^{2}$ = 0.469, indicating the usual finding of higher error rates for saccades vs. manual responses (14.8% vs. 8.0%, see, e.g., Huestegge and Koch, 2009, 2010, 2013). There was also a significant main effect of response condition, *F*(1,21) = 6.99, *p* = 0.015, $\eta_{p}^{2}$ = 0.250 (single: 8.8%, dual: 13.9%), and a significant main effect of the number of S–R rule sets, *F*(1,21) = 33.84, *p* < 0.001, $\eta_{p}^{2}$ = 0.617 (one S–R rule: 6.1%, two S–R rules: 16.6%). There were no significant (two-way/three-way) interactions with respect to error rates (all *F*s < 1 except for the response condition^∗^number of S–R rule sets interaction: *F*(1,21) = 1.99, *p* = 0.174). Taken together, the error analysis shows that the interpretation of the effects of the number of S–R rule sets on RTs is in no way compromised by any speed-accuracy trade-offs.

**Table 3:** Error rates (%) for manual responses and saccades in the two-rule sets condition as a function of task condition (single vs. dual) and rule transition (repetition vs. switch) in Experiment 2.

	Single response condition		Dual response condition	
	Repetition	Switch	Repetition	Switch
Manual responses	8.1 (2.0)	11.2 (2.6)	13.6 (3.6)	22.1 (3.3)
Saccades	13.4 (3.5)	18.6 (3.5)	24.6 (4.3)	25.4 (4.0)

*Numbers in parentheses denote standard errors.*

#### Rule Transition Effects

**FIGURE 4:**
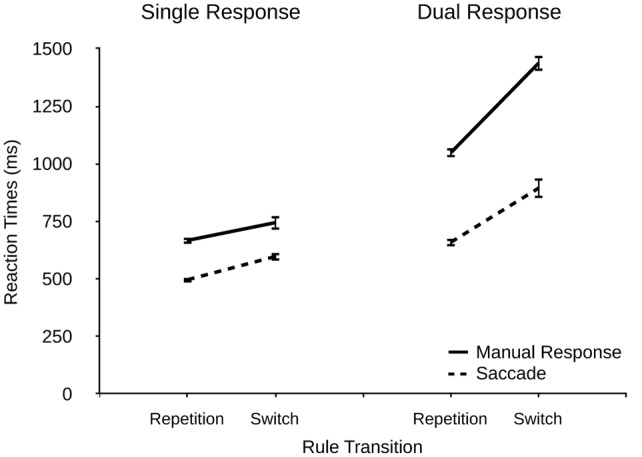
**Reaction times (ms) for manual responses and saccades as a function of rule transition (repetition and switch) and response condition (single and dual) in the two-rule condition in Experiment 2.** Error bars denote standard errors.

Additionally, we analyzed the data of the two-rule condition in more detail as a function of local rule transitions (rule repetitions vs. rule switches), response modality (manual response vs. saccade), and response condition (single vs. dual). If our assumption of additional rule retrieval processes in conditions involving two rules is correct, we should observe corresponding performance costs for rule switches. RTs are shown in **Figure 4**.

We found a significant main effect of rule transition, *F*(1,21) = 77.62, *p* < 0.001, $\eta_{p}^{2}$ = 0.779, with *M* = 919 ms for switches vs. *M* = 718 ms for repetitions. This result demonstrates that task rule switches affected performance, most likely reflecting interference due to retrieving or activating the task-relevant rule (or inhibiting the task-irrelevant rule). Furthermore, there were significant main effects of modality, *F*(1,21) = 177.74, *p* < 0.001, $\eta_{p}^{2}$ = 0.890 (manual responses: 975 ms, saccades: 662 ms) and response condition, *F*(1,21) = 121.21, *p* < 0.001, $\eta_{p}^{2}$ = 0.846 (single: 627 ms, dual: 1011 ms).

There was a significant interaction of modality and response condition, *F*(1,21) = 64.48, *p* < 0.001, $\eta_{p}^{2}$ = 0.746, indicating larger dual-response costs for manual responses (537 ms) than for saccades (231 ms), and a significant interaction of modality and rule transition, *F*(1,21) = 6.78, *p* = 0.016, $\eta_{p}^{2}$ = 0.236, showing larger rule switching costs for manual responses (234 ms) than for saccades (167 ms). Interestingly, the interaction of response condition and rule transition was also significant, *F*(1,21) = 28.50, *p* < 0.001, $\eta_{p}^{2}$ = 0.564, indicating that rule switching costs were larger in dual-response conditions (312 ms) that in single-response conditions (89 ms). This finding reveals that rule retrieval interfered with dual-response coordination efficiency in that rule retrieval elevated dual-response costs in switch trials compared to repetition trials. This result was further qualified by a three-way interaction, *F*(1,21) = 14.27, *p* = 0.001, $\eta_{p}^{2}$ = 0.393, showing that rule switching costs were similar in single-response conditions for both response modalities (manual responses: 77 ms, saccades: 101 ms) while they differed pronouncedly in dual-response conditions (manual responses: 389 ms, saccades: 235 ms), thus resembling the results from the main analysis which indicated larger interference effects for manual responses.

There was a significant effect of response modality on error rates, *F*(1,21) = 12.13, *p* = 0.002, $\eta_{p}^{2}$ = 0.355, indicating the typical finding of higher error rates for saccades than for manual responses (20.5% vs. 13.8%). There was also a significant main effect of response condition, *F*(1,21) = 6.69, *p* = 0.017, $\eta_{p}^{2}$ = 0.233 (single: 12.8%, dual: 21.4%), and a significant main effect of rule transition, *F*(1,21) = 14.42, *p* = 0.001, $\eta_{p}^{2}$ = 0.396, with overall switching costs of 4.4% points (19.3% vs. 14.9%). There were no significant two-way interactions (all *F*s < 1 except for the modality^∗^transition interaction: *F*(1,21) = 2.587, *p* = 0.122). However, the three-way interaction was significant, *F*(1,21) = 8.05, *p* = 0.010, $\eta_{p}^{2}$ = 0.268, suggesting that switching costs differed more pronouncedly between response modalities in dual-task conditions (switching costs of 8.5% for manual responses and 0.8% for saccades) than in single-task conditions (manual responses: 3.1%, saccades: 5.2%). Taken together, the error analysis shows that the interpretation of the switching effects on RTs is in not compromised by any speed-accuracy trade-offs.

## General Discussion

The present study addressed two important research questions in the domain of multiple-action control in order to address the interplay of action control and mechanisms of maintenance and retrieval in working memory: In Experiment 1, we studied the impact of the number of task-relevant binding patterns while keeping the number of instructed S–R rule sets constant, whereas in Experiment 2 we studied the impact of the number of task-relevant S–R rule sets while keeping the number of task-relevant binding patterns constant. Based on previous theory, we hypothesized that both manipulations should lead to retrieval competition (either between relevant response bindings or S–R rule sets), which should not only elevate overall performance (in terms of increased RTs and/or error rates) but more specifically affect dual-response coordination efficiency, which is reflected in the amount of dual-response costs (i.e., the difference between dual- and single-response performance). The present research questions were addressed in a setting involving the combination of oculomotor and manual responses.

As a main result, we found that indeed dual-response coordination efficiency (as indicated by dual-response costs in RTs) was affected by retrieval competition regarding both the number of task-relevant binding patterns (Experiment 1) and the number of task-relevant S–R rule sets (Experiment 2). A numerical comparison of effect sizes suggests that the higher-level (rule-based) manipulation in Experiment 2 had a much stronger effect than the more basic (response combination-based) effect in Experiment 1, suggesting that it is easier for participants to cope with an increase of response complexity within one common rule than with an increase in the number of task rules (despite the same amount of possible responses in the one-rule vs. two-rule condition). Specifically, the analysis of local rule switch effects in Experiment 2 suggested that dual-response performance (and not only response times in general) suffered substantially after rule switches (compared to rule repetitions), indicating that rule retrieval specifically affected dual-response coordination.

The present results extend previous theoretical claims in the dual-task control literature. For example, Ellenbogen and Meiran (2008) claimed that dual-task costs might be attributed to conflicts that arise when during the execution of one task rules for another task need to be held active in memory in order to enhance preparation for the latter. This can, for example, yield parallel activation of response codes, which can thus interfere and produce crosstalk phenomena (see also Hommel, 1998a; Hommel and Eglau, 2002). Our present Experiment 1, which represents a typical dual-task experiment (i.e., in form of a manual and a saccade task triggered by separate stimuli), shows that even when the instructed task rules are held constant (i.e., for both effector systems participants were instructed to initiate a response which is spatially compatible with the stimulus), the actual size of the set of task-relevant response binding patterns plays an additional important role in determining the efficiency of task coordination, even across highly different response modalities such as manual and oculomotor responses. Experiment 2 also extends the claims of rule-based conflict as a major determinant of dual-response control efficiency by showing that S–R rule-based conflict not only affects dual-response performance in typical dual-task settings (i.e., with two separate stimuli as in Experiment 1), but also when response compounds are triggered by a single dimension of a stimulus. Note that manipulating the number of S–R rule sets by utilizing opposing S–R mappings for the two rules in Experiment 2 was also associated with dimensional overlap between S–R rule sets (i.e., responses were bivalent). Therefore, the observed effects might not only have occurred due to the different number of S–R rule sets alone, but might additionally be based on response-based conflict across trials (e.g., Meiran, 2000; Brass et al., 2003). Therefore, future research could explore effects of the number of S–R rule sets in the absence of potential interference between S–R rule sets. Taken together, the present results represent a step forward in understanding the interaction of memory and action selection in the context of multiple-action control.

Our observations, especially those in Experiment 1, further strengthen our proposed framework of multiple-action control (Huestegge and Koch, 2010, see introduction) by confirming a prediction that was not explicitly tested previously, namely that the number of task-relevant binding patterns affects the efficiency of the coordination of two responses during action selection (as indexed by the amount of dual-response costs). This result further emphasizes that any model of dual-task control that only focuses on mechanisms within a trial (e.g., Pashler, 1994; Meyer and Kieras, 1997; Navon and Miller, 2002; Tombu and Jolicoeur, 2003) is necessarily incomplete, since contextual factors (e.g., which binding patterns are required in surrounding trials) strongly determine action selection efficiency (see also Janczyk, 2016). Our observation of rule switch costs further strengthens this claim by demonstrating that maintaining two rules in working memory affected dual-response efficiency on a trial-by-trial basis. The present results further highlight that it is important to study the interaction of action control and memory in order to specify the largely elusive notion of “response selection” in dual-task control models.

An interesting additional observation in Experiment 1 was that the number of response alternatives did not strongly affect overall RTs (Hick’s law) in oculomotor control. While it should be noted that the underlying group comparison might be less powerful than a within-subjects design, the absence of Hick’s law for oculomotor responses is well in line with previous studies (e.g., Kveraga et al., 2002). Interestingly, although for manual responses the number of response alternatives did not differ between groups (thus no effect in single-response conditions was expected) we found a strong modulation of manual (but not oculomotor) dual-response costs when the number of alternatives in the oculomotor response varied, thus indicating a decrease of response coordination efficiency with a larger number of binding patterns. This result pattern suggests that it is important to analyze measures of coordination costs and not only overall RT levels when studying the impact of response alternatives in the context of multiple-action control. Additionally, the potential dependency of underlying mechanisms on the specific effector systems involved calls for further research utilizing other combinations of output systems (e.g., Huestegge and Hazeltine, 2011; Stephan et al., 2013; Huestegge et al., 2014).

In sum, the present study on cross-modal multiple action control demonstrated that retrieval competition between task-relevant response binding patterns and task rules both have a strong impact on complex action control. These results further highlight the importance of studying the interplay of memory and action control (here: retrieval competition) in order to specify the mechanisms underlying the rather vague notion of response selection in multiple action control.

## Ethics Statement

All procedures performed in studies involving human participants were in accordance with the ethical standards of the national research committee of the DGPs (Deutsche Gesellschaft für Psychologie) with written informed consent from all subjects. All subjects gave written informed consent in accordance with the Declaration of Helsinki (1964). The protocol was approved by the Deutsche Forschungsgemeinschaft (DFG) within the grant HU 1847/3-1. This article does not contain any studies with animals performed by any of the authors.

## Author Contributions

LH had the idea for the study. AP and LH conjointly designed experiments and wrote and revised the manuscript. AP programmed the experiments and analyzed the data.

## Conflict of Interest Statement

The authors declare that the research was conducted in the absence of any commercial or financial relationships that could be construed as a potential conflict of interest.
