# Supplementary material for: Cross-modal Action Complexity: Action- and Rule-related Memory Retrieval in Dual-response Control
Source: Front Psychol. 2017 Apr 7;8:529. doi: 10.3389/fpsyg.2017.00529 (PMC5383708; doi:10.3389/fpsyg.2017.00529)
Supplement: Supplementary file 1 [file Data_Sheet_1.docx]

Appendix

Compatibility effects in Experiment 1

Since in the main analysis only incompatible trials were included, we additionally report *compatibility effects* (see Figure A1) by including also compatible trials (note that in the current design responses were compatible only in one fourth of the trials). We computed the same ANOVAs as described in Experiment 1, however, with compatibility as an additional factor in addition to response condition and number of binding patterns. For the sake of brevity, we here only focus on the main effects of compatibility and the interactions of compatibility and response condition to assess how compatibility affected mean RTs and dual-response costs, respectively. Finally, we also tested whether the crucial interaction effect in manual RTs reported above was modulated by compatibility (i.e., whether there was a significant three-way interaction).

*Effects of compatibility on overall RTs and error rates*. There was a significant main effect of compatibility on manual RTs, *F*(1, 45) = 146.76, *p* < .001, η_p_² = 765, indicating longer manual RTs in incompatible vs. compatible conditions (769 ms vs. 658 ms). The same effect was found in manual error rates, *F*(1, 45) = 6.45, *p* = .015, η_p_² = .125 (6.1% vs. 4.0%), saccade RTs, *F*(1, 45) = 68.34, *p* < .001, η_p_² = .603 (459 ms vs. 404 ms), and saccade error rates, *F*(1, 45) = 8.86, *p* = .005, η_p_² = .164 (7.3% vs. 3.0%). Thus, incompatibility negatively affected overall performance in both the manual and oculomotor domain.

*Effects of compatibility on dual-response costs.* There was a significant interaction of compatibility and response condition for manual RTs, *F*(1, 45) = 265.28, *p* < .001, η_p_² = 855 with dual-response costs of 314 ms for compatible responses and 562 ms for incompatible responses, for manual errors, *F*(1, 45) = 51.31, *p* < .001, η_p_² = .533 (dual-response costs of -2.6% for compatible vs. dual-response costs of 7.0% for incompatible responses), for saccade RTs, *F*(1, 45) = 74.03, *p* < .001, η_p_² = .622 (dual-response costs of 95 ms for compatible and 204 ms for incompatible responses), and saccade errors, *F*(1, 45) = 81.90, *p* < .001, η_p_² = 645 (0.0% in compatible vs. 10.8% for incompatible responses). Thus, incompatibility increased dual-response costs in both the manual and oculomotor domain. There was no significant three-way interaction of response condition, the number of response patterns, and

compatibility, neither for manual RTs, *F*(1, 45) = 2.36, *p* = .132, nor for manual response errors, *F* < 1, and neither for saccade RTs nor saccade errors, both *F*s < 1. Thus, the important effect of the number of task-relevant response patterns on dual-response costs was not significantly affected by compatibility.

This observation of compatibility effects on both overall RTs and dual-response costs extends previous reports of compatibility effects across saccades and manual responses in the context of two-choice responses (e.g., Huestegge & Koch, 2009). However, it is important to note that compatibility not only affected overall RT levels, but also dual-response costs indicating response coordination efficiency (see also the related notion of crosstalk, e.g., Miller, 2006; Navon & Miller, 1987).

*Figure A1.* Dual-response costs (ms) for manual response and saccades in Experiment 1 as a function of number of R-R compatibility (compatible vs. incompatible) and response binding patterns (constrained vs. unconstrained), which are calculated here as the RTs increase for dual-response trials relative to single-response RTs.

Table A1. *Error rates (%) for manual responses and saccades in Experiment 1 as a function of response-response compatibility (compatible vs. incompatible), number of response binding patterns (constrained vs. unconstrained), and response condition (single and dual). Numbers in parentheses denote standard errors.*

|  | R-R Compatible | | | | | | | R-R Incompatible | | | | | | |
| --- | --- | --- | --- | --- | --- | --- | --- | --- | --- | --- | --- | --- | --- | --- |
| Number of Patterns | Constrained | | | Unconstrained | | | Constrained | | | | Unconstrained | | |  |
| Response Condition | Single | Dual | Single | | Dual | Single | | | Dual | Single | | Dual |  |  |
| Manual Responses | 6.0 (1.5) | 3.6 (1.3) | 4.7 (1.5) | | 1.7 (1.3) | 2.9 (0.7) | | | 10.3 (1.5) | 2.3 (0.7) | | 8.8 (1.6) |  |  |
| Saccades | 5.2 (2.5) | 4.0 (1.5) | 0.8 (2.5) | | 1.9 (1.5) | 2.1 (0.4) | | | 11.8 (1.4) | 1.7 (0.5) | | 13.5 (1.5) |  |  |
